# Transcriptional networks are associated with resistance to *Mycobacterium tuberculosis* infection

**DOI:** 10.1371/journal.pone.0175844

**Published:** 2017-04-17

**Authors:** Chetan Seshadri, Nafiseh Sedaghat, Monica Campo, Glenna Peterson, Richard D. Wells, Gregory S. Olson, David R. Sherman, Catherine M. Stein, Harriet Mayanja-Kizza, Ali Shojaie, W. Henry Boom, Thomas R. Hawn, on behalf of the Tuberculosis Research Unit (TBRU)

**Affiliations:** 1 Department of Medicine, University of Washington, Seattle, Washington, United States of America; 2 Computer Engineering School, Iran University of Science and Technology, Tehran, Iran; 3 Center for Infectious Disease Research, Seattle, Washington; 4 Department of Epidemiology & Biostatistics, Case Western Reserve University, Cleveland, Ohio, United States of America; 5 Makerere University, Kampala, Uganda; 6 Department of Biostatistics, University of Washington, Seattle, Washington, United States of America; 7 Department of Medicine, Case Western Reserve University, Cleveland, Ohio, United States of America; Rutgers Biomedical and Health Sciences, UNITED STATES

## Abstract

**Rationale:**

Understanding mechanisms of resistance to *M*. *tuberculosis* (M.tb) infection in humans could identify novel therapeutic strategies as it has for other infectious diseases, such as HIV.

**Objectives:**

To compare the early transcriptional response of M.tb-infected monocytes between Ugandan household contacts of tuberculosis patients who demonstrate clinical resistance to M.tb infection (cases) and matched controls with latent tuberculosis infection.

**Methods:**

Cases (n = 10) and controls (n = 18) were selected from a long-term household contact study in which cases did not convert their tuberculin skin test (TST) or develop tuberculosis over two years of follow up. We obtained genome-wide transcriptional profiles of M.tb-infected peripheral blood monocytes and used Gene Set Enrichment Analysis and interaction networks to identify cellular processes associated with resistance to clinical M.tb infection.

**Measurements and main results:**

We discovered gene sets associated with histone deacetylases that were differentially expressed when comparing resistant and susceptible subjects. We used small molecule inhibitors to demonstrate that histone deacetylase function is important for the pro-inflammatory response to *in-vitro* M.tb infection in human monocytes.

**Conclusions:**

Monocytes from individuals who appear to resist clinical M.tb infection differentially activate pathways controlled by histone deacetylase in response to in-vitro M.tb infection when compared to those who are susceptible and develop latent tuberculosis. These data identify a potential cellular mechanism underlying the clinical phenomenon of resistance to M.tb infection despite known exposure to an infectious contact.

## Introduction

Despite the availability of cost-effective drugs and a safe vaccine, *Mycobacterium tuberculosis* (M.tb) was responsible for over 1.5 million deaths worldwide in 2014[1]. Understanding mechanisms of pathogenesis could lead to the development of more effective interventions. Animal studies have revealed the importance of IFN-γ and TNF- α for controlling mycobacterial replication[2–4]. These are supplemented by studies of humans who are hypersusceptible to mycobacterial infection as a result of rare genetic mutations in IFN-γ signaling pathways or pharmacologic blockage of TNF-α[5]. Further, co-infection with HIV has emerged as a major reason for the resurgence in tuberculosis, and this effect is not purely due to T-cell depletion[6–10]. Collectively, these studies have only uncovered a partial understanding of the mechanisms underlying susceptibility to mycobacterial infection and disease.

Historically, significant breakthroughs have emerged by studying mechanisms of resistance to infections. A contemporary example is protection of individuals with CCR5Δ32 from HIV infection[11,12]. This discovery led directly to the development of CCR5 inhibitors as drugs[13]. With respect to tuberculosis, individuals may resist initial infection with M.tb or resist the progression from infection to disease. However, mechanisms of resistance to M.tb infection are difficult to study for a number of reasons. First, the diagnosis of M.tb infection is based on an immune response to M.tb proteins rather than direct microbiologic confirmation because there is no test that measures the presence of M.tb *in-vivo*. Second, because it is an airborne disease, exposure is difficult to quantify and requires extensive epidemiologic investigation. Nevertheless, there have been some attempts to identify individuals who might be able to resist clinical M.tb infection. In the pre-antibiotic era with epidemic rates of tuberculosis in the population, some individuals never demonstrated a positive tuberculin skin test (TST), including workers at tuberculosis hospitals[14]. A genetic linkage study in South Africa revealed a locus on chromosome 11p14 that was associated with a lack of TST reactivity[15].

More recently, our group established a longitudinal cohort in Uganda in which we identified household contacts of tuberculosis patients and followed them prospectively[16,17]. Among 2585 household contacts of 872 index cases, we identified 255 (9.9%) that were persistently TST negative on repeated testing over at least two years of follow-up. These individuals also did not develop active disease during this time. We hypothesized that there might be a genetic basis to this phenotype, and published a genome-wide linkage study in which we identified two chromosomal loci associated with a negative TST[18]. Even though the TST reflects T-cell memory to mycobacterial antigens, the genetic associations suggest host-intrinsic factors may govern TST conversion after exposure. These data led us to consider whether there might be functional differences in the cellular response to *in-vitro* M.tb infection between these two clinical groups. Here, we conducted a comparative transcriptomic study and identified differentially expressed gene sets associated with a persistently negative TST. These data revealed that a cellular pathway involving inhibition of histone deactylase is selectively induced among individuals with apparent clinical resistance to M.tb infection.

## Materials and methods

### Clinical cohort

We previously published full details of the Kawempe Community Health Study[16,17]. Briefly, newly diagnosed tuberculosis patients were identified at the Uganda National Referral Tuberculosis Treatment Center at Upper Mulago Hospital in Kampala, Uganda. The index cases were enrolled if they had culture confirmed pulmonary tuberculosis and had at least one household contact living with them[19]. Between 2002 and 2012, 2585 household contacts were enrolled and followed prospectively for up to two years for development of tuberculosis disease or diagnosis of latent tuberculosis infection by serial TSTs at 0, 3, 6, 12, 18,and 24 months. This study did not include M.tb-specific interferon gamma release assays (IGRA) because these were not commercially available at the onset of this study. Among all household contacts, 28.5% (N = 737) were TST negative at the initial visit and 34.5% of this group (N = 255) remained TST negative over two years of follow-up. For this study, we define subjects with a persistently negative TST as ‘cases’ and subjects with a positive TST as ‘controls.’ We obtained cryopreserved peripheral blood mononuclear cells (PBMC) obtained at enrollment from a convenience samples of 22 cases and 30 controls based on the availability of PBMC for the proposed studies. Demographic and clinical characteristics are shown in Table 1. All subjects were HIV-uninfected. Accumulated epidemiologic risk was calculated using a method originally developed for children under 15 and an adapted version for adults over age 15[20,21]. Because only five individuals were less than 15 years old in this analysis, we report only the adult risk scores. Evidence of past BCG vaccination was based on presence of a characteristic scar. BMI was calculated based on weight and height upon enrollment.

**Table 1:** Demographic and clinical description of study cohort.

	cases	controls	p-value
N	14	22	
Female (%)	10 (71%)	16 (72%)	0.930
Age, Median (IQR)	16 (13–20)	27.5 (19–32)	0.025
Presence of BCG scar	9 (64.3%)	15 (68.2%)	0.517
Epidemiologic risk score^a^, Median (range)	6.0 (5–9)	6.5 (5–9)	0.369
BMI, Median (IQR)	21.6 (18.3–24.2)	23.3 (21.1–25.8)	0.229

^a^Calculated for individuals age ≥ 15 only, using the method in Ma et al. 2014, because there were too few individuals < 15 years old

### In-vitro infection and RNA extraction

Archived PBMC were thawed and washed twice with RPMI supplemented with 10% fetal calf serum (Atlas Biologicals). Cell viability as determined by trypan blue staining was greater than 80%. Monocytes expressing CD14 were isolated by positive selection (Miltenyi). Depending on the yield, between 500,000 and one million monocytes were plated in duplicate in a 24-well tissue culture plate and rested overnight at 37°C, 5% CO_2_. The next day, cells were mock-infected or infected with virulent *M*. *tuberculosis* strain H37Rv at a multiplicity of infection (MOI) 10:1. After a six-hour incubation at 37°C, 5% CO_2_, the total RNA was harvested using TRIzol-LS (Invitrogen). We did not observe significant toxicity at this early time point (data not shown). TRIzol samples were stored at -80°C until all infections were completed. To minimize batch effects, equal numbers of cases and controls were processed on each of six days and frozen aliquots of M.tb were used for infections.

TRIzol samples were thawed and the contents transferred to Phase Lock Gel tubes (5PRIME GmbH) and supplemented with 0.2 ml chloroform (Fisher Scientific). Phase Lock Gel tubes were spun at 12,000g for 15 minutes at 4°C and the upper aqueous layer transferred to a 2ml Eppendorf tube. An equal volume of 70% ethanol was added dropwise and mixed by tapping. The contents of this Eppendorf tube were then processed using RNeasy Mini Kit (Qiagen) according the manufacturer’s instructions with the following modifications. DNA digestion was performed with DNAse I (Qiagen) and the final elution was performed twice with 30ul RNase-free water to maximize the yield. Subsequently, RNA was dried down in a Speedvac and resuspended in 10ul water before assessing purity, integrity, and yield. The average RNA Integrity Number (RIN) as measured by Bioanalyzer was 8 and the average yield as measured by Nanodrop was 153 ng.

### Microarrays data processing and analysis

Total RNA (50-100ng) was transcribed into complementary DNA, labeled, and hybridized onto Illumina HT-12 v3 microarrays according to the manufacturer’s instructions. This platform includes 48,804 probes covering more than 20,000 annotated genes and 12,000 expressed sequence tags. The initial dataset consisted of 13 case and 22 control subjects, each with paired microarrays from media-treated or M.tb-treated monocytes. Thus, a total of seventy RNA samples were submitted to the Genomics Core at the Fred Hutchinson Cancer Research Center in Seattle, WA. Four samples did not meet pre-specified quality control criteria after labeling so sixty-six samples underwent hybridization. Principal components analysis revealed a batch effect among ten samples, so these were removed from downstream analysis (S1 Fig). Thus, the final discovery dataset consisted of 10 case and 18 control subjects. Full details regarding data processing and expression analysis, including Limma, Signaling Pathway Interaction Analysis (SPIA), and Gene Set Enrichment Analysis (GSEA), are described in Supplementary Methods[22–25]. SPIA and GSEA examined the enrichment of 137 and 4726 gene sets, respectively. The data discussed in this publication have been deposited in NCBI's Gene Expression Omnibus (GEO) in MIAME-compliant format and are accessible through GEO Series accession number GSE76873[26–28].

### Interaction networks

To delineate interactions among enriched pathways, genes in all enriched pathway, as well as interactions among genes, were extracted from KEGG using the ‘graphite’ package in R[29]. Pathways were considered enriched in either cases and controls samples if they were found significant at FDR 10% by SPIA because of the small number of pathways considered by this method. FDR threshold was set at 20% for GSEA for the purposes of hypothesis generation and in the spirit of the original publications [24]. Connectedness between pairs of pathways was defined based on the number of genes common between both pathways. To quantify the overlap between pathways, we used the Jaccard Index, i.e. the ratio of the number of genes common between the two pathways over the total number of genes in both pathways. To obtain an interpretable network, an edge between a pair of pathways was drawn if the Jaccard index was at least 0.1.

### Cellular experiments

Human monocytes were isolated from peripheral blood of healthy volunteers by Ficoll gradient separation followed by positive selection using human anti-CD14 antibody assisted magnetic bead separation (Miltenyi Biotec, Auburn, CA). Human pro-monocytic cell line U937 (American Type Culture Collection, ATCC CRL-1593.2) and isolated monocytes were maintained in cell culture at 37°C and 5% CO2 in RPMI 1640 medium containing 10% FCS. Cells were pretreated with 4-phenylbutyrate (Tocris), sodium butyrate (Sigma), or depsipeptide (Sigma) for 1 hour and then stimulated with LPS (List Biological) and M.tb whole cell lysate (BEI resources) in the case of U937s or live M.tb strain H37Rv (D. Sherman) at a multiplicity of infection (MOI) of 2.5 in the case of monocytes for 24 hours. These MOIs were optimized in preliminary experiments to balance the production of inflammatory cytokines and cellular toxicity (data not shown). Subsequently, supernatants were harvested and cytokines (IL-6, TNF, IL-1β) quantified by ELISA (R&D Systems, Minneapolis, MN). Each sample was assayed in duplicate, and experiments shown were performed at least twice to ensure reproducibility. Phenylbutyrate and sodium butyrate were dissolved in water, and depsipeptide was dissolved in DMSO.

### Ethics approval

The institutional review boards (IRB) at University Hospitals of Cleveland Medical Center and the Uganda Council for Science and Technology approved the Ugandan study. All individuals in the Ugandan study provided written informed consent, including written consent from the parents of study participants. The lab-based investigations described here were further approved by the IRB at the University of Washington.

### Bacterial growth assay

M.tb strain Erdman was transformed with vector encoding the bacterial luciferase operon, and frozen stocks were generously provided Dr. Jeffery Cox (UCSF). We confirmed a linear relationship between relative light units (RLU) and colony forming units (CFU) (data not shown). To analyze the effect of phenylbutyrate, sodium butyrate, depsipeptide, and rifampin (final concentration of 0.1 ug/ml) in broth culture, bacterial stock cultures were thawed and diluted to an OD600 of 0.005 in 7H9+GAT media containing Difco Middlebrook 7H9 Broth (BD) containing BBL Middlebrook ADC enrichment (BD), 0.05% Tween 80 (Sigma), and 0.2% Glycerol. Next, we made serial dilutions of each drug to a final concentration of 10X in 7H9+GAT supplemented with 10% DMSO. In a 96-well U-bottom tissue culture plate (Falcon), 180 ul of the diluted bacterial culture was added to each well and 20 ul of each drug was added at 1/10 volume (in duplicate). For the 1% (MIC 99) control, 200 ul of diluted bacterial culture was added to each well. Plates were incubated at 37C° 5% CO_2_ for 8 days at which time cultures were transferred to 96-well white well plates (Nunc, 136101) and relative light units (RLUs) were measured using a plate reader (Synergy H4, BioTek).

## Results

### The early transcriptional response to in-vitro M.tb infection is broadly conserved among cases and controls

**Fig 1:**
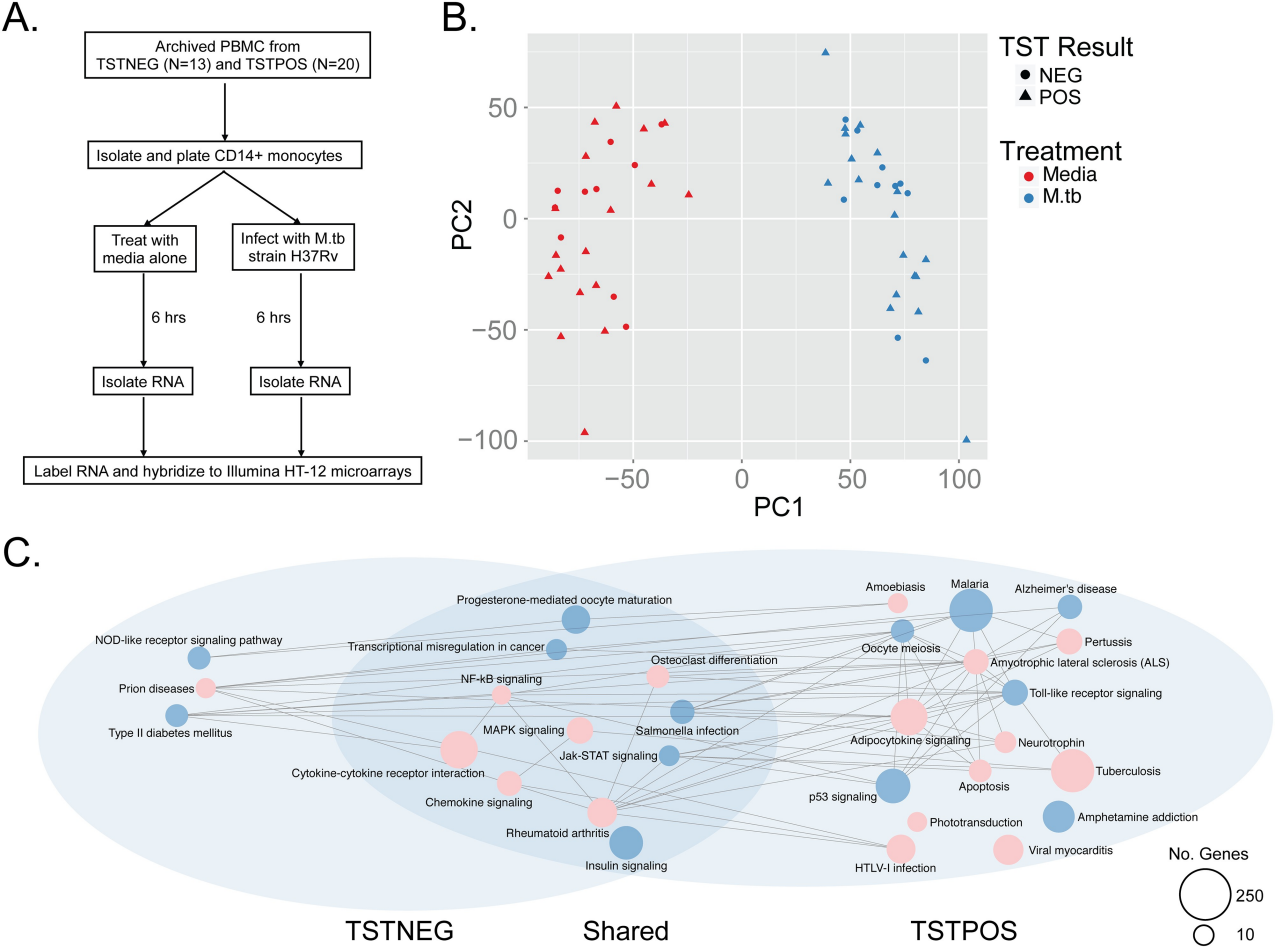
The early monocyte transcriptional response to M.tb infection. (A) Schematic of study design. CD14+ monocytes from cases or controls were isolated from cryopreserved PBMC and exposed to either media or M.tb strain H37Rv for six hours prior to harvesting total RNA for transcriptional profiling with Illumina HT-12 microarrays. (B) Principal components analysis was performed on quantile normalized data and reveals M.tb infection as the major source of variability in the data. (C). Network diagram generated from KEGG pathways identified through Signaling Pathway Interaction Analysis (SPIA) reveals that largely overlapping cellular processes are perturbed after infection. The color of each node represents the status of the pathway as either activated (red) or inhibited (blue). The size of the node is proportional to the number of genes in the pathway.

We isolated peripheral blood monocytes from 10 cases and 18 controls, infected them with live M.tb for six hours, and measured genome-wide transcriptional profiles (Fig 1a and Table 1). Principal components analysis revealed that infection was the greatest source of variability in the data, accounting for 40% in PC1, and this was much greater than any technical variable, such as batch, RNA quality, or RNA yield (Fig 1b and data not shown). Notably, TST status did not differentiate samples within the first two principal components (Fig 1b). Among TST-negative subjects we observed 3490 induced and 4543 suppressed genes after M.tb infection with FDR 10%. Among TST-positive subjects we observed 4199 induced and 5408 suppressed genes after M.tb infection with FDR 10%. We then used Signaling Pathway Impact Analysis (SPIA) to investigate whether these genes represented canonical pathways that were differentially activated or suppressed (perturbed) between cases and controls subjects[23]. The advantage of SPIA is the ability to take into account the natural topology of a biological network by simultaneously accounting for genes that are both induced and suppressed in the same pathway. We found 12 KEGG pathways that were significantly perturbed among control subjects, and a subset of these was also perturbed among cases (S2 Fig). We generated network graphs using pathways that were significantly enriched at FDR 10% (Fig 1c). We found that a core group of 11 highly connected biological processes are similarly perturbed in monocytes derived from both cases and controls. Additionally, 16 pathways were preferentially perturbed among controls and three pathways were specifically perturbed among cases. A closer review of the data revealed that some of these groups-specific pathways were also perturbed in the other group but at an FDR cut-off just beyond the threshold value of 10% (data not shown). Taken together, these data reveal that the early transcriptional response to in-vitro M.tb infection is highly conserved but some processes may be associated with either a positive or negative TST among tuberculosis household contacts in Uganda.

### Gene set enrichment analysis reveals pathways uniquely associated with resistance to clinical M.tb infection

**Fig 2:**
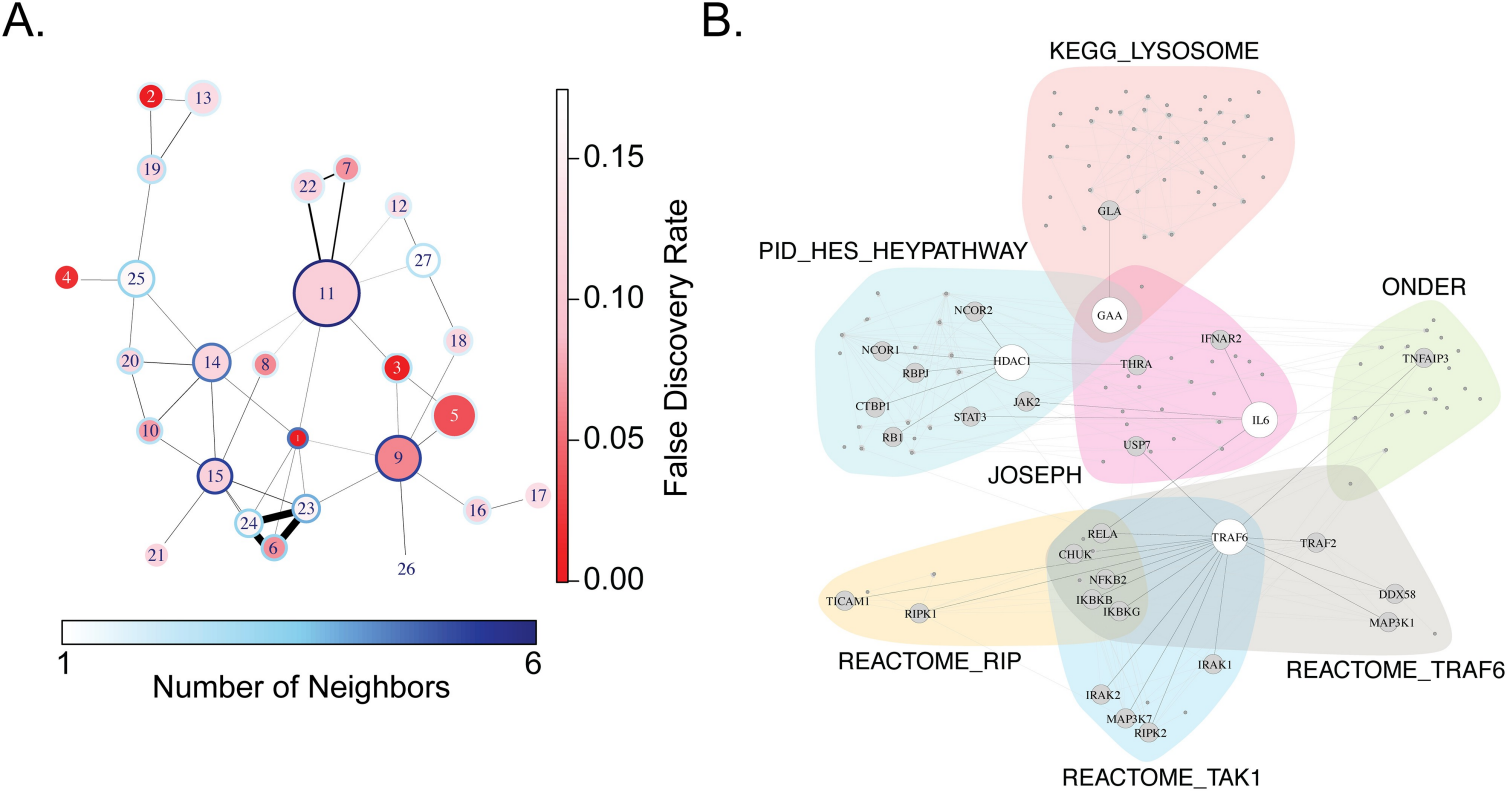
Network analysis of gene sets unique to cases demonstrating resistance to M.tb infection. (A) A network diagram was generated from the results of GSEA listed in Table 2 with a FDR < 20%. In this network, each node corresponds to a gene set (pathway); an edge is drawn between two gene sets with a black line if the genes in the two gene sets overlap. Here, the diameter of each node corresponds to the relative number of genes in each gene set; the degree of blue color shading in the perimeter of each node represents the number of its neighbors (number of gene sets whose members overlap); the degree of central red color shading reflects significance (FDR value); and the thickness of each edge corresponds to the number of genes shared by the two neighboring gene sets (proportional to the Jaccard Index between pathways). Gene set 1 is ‘JOSEPH_RESPONSE_TO_SODIUM_BUTYRATE.’ (B) Venn diagram centered on the ‘JOSEPH’ gene set and its 6 neighboring gene sets in diagram. Each shaded area corresponds to a gene set, and edges between pairs of genes correspond to gene-gene interactions defined in KEGG, obtained using the ‘graphite’ R-package. Genes present in the gene set but absent from the KEGG database are not shown for clarity. The location of HDAC1, GAA, TRAF6, and IL-6 are highlighted along with their neighbors.

To expand our analysis, we collapsed the data by subtracting background values of gene expression in media control wells to compare fold changes in gene expression after in-vitro M.tb infection between cases and controls. We found 210 genes that were differentially expressed at a nominal p-value of 0.05; however, after correcting for multiple hypothesis testing using the Benjamini-Hochberg method, none of these were statistically significant (data not shown). This highlights the limitations of a gene-by-gene approach in an experimental setting where we have a priori knowledge that a substantial fraction of observed changes in gene expression are shared between the two groups (Fig 1c). To expand to a gene-set based analysis not just limited to KEGG pathways, we employed Gene Set Enrichment Analysis (GSEA), which has the additional advantage of dichotomizing the background-subtracted data according to TST status, which is our variable of interest. Using GSEA, we found 24 and 27 gene sets uniquely associated with controls and cases at FDR 20%, respectively (Table 2 and S1 Table). The knowledge base accessed by GSEA includes both experimentally derived as well as manually curated gene sets that are often overlapping, so we constructed an interaction network of the gene sets enriched among cases. The degree of overlap, the size and significance of the pathways, and the number of their neighboring pathways are depicted in Fig 2a. The most significantly enriched (gene set 1) among cases subjects is ‘JOSEPH_RESPONSE_TO_SODIUM_BUTYRATE_DN,’ a study of the effects of sodium butyrate on gene expression in a lung cancer cell line. We constructed a Venn Diagram of the subnetwork centered on this gene set to highlight the biological processes and gene-gene interactions. Notably, we found that ‘JOSEPH’ interacted with innate inflammatory signaling through well-known mediators, such as IL-6 and TRAF6 (Fig 2b). ‘JOSEPH’ also interacted with lysosomal function through its effects on alpha-glucosidase (GAA). We also found a second HDAC-related gene set that was enriched among cases (PEART_HDAC-PROLIFERATION_CLUSTER_UP, FDR 0.036). To confirm the robustness of these associations, we performed a sensitivity analysis in which we included the ten samples that were removed because of batch effects (S1 Fig). We found 26 gene sets enriched among cases of which seven overlapped with our primary results, including ‘JOSEPH’ (S1 Table). We also performed network analysis using the EnrichmentMap Cytoscape App and results were identical to that shown in Fig 2A (data not shown) [30]. Finally, we found cellular processes associated with HDAC function in gene sets associated with controls, suggesting that these functions may be central for the innate immune response to mycobacteria (DELLA_RESPONSE_TO_TSA_AND_BUTYRATE AND PID_HDAC_CLASSII_PATHWAY) (S2 Table). These analyses suggest that a set of biological processes centered on the effects of HDACi on cells are differentially expressed in monocytes when comparing M.tb-resistant and M.tb-susceptible individuals after *in-vitro* infection with M.tb.

**Table 2:** Gene sets preferentially associated with cases demonstrating resistance to M.tb infection.

RANK	NAME	SIZE	NES	FDR
1	**JOSEPH_RESPONSE_TO_SODIUM_BUTYRATE_DN** Genes suppressed in H460 cells (non-small cell lung cancer) after sodium butyrate treatment	37	-2.34	0.001
2	KEGG_TYROSINE_METABOLISM Canonical pathway for metabolism of tyrosine	17	-2.33	0.000
3	PATTERSON_DOCETAXEL_RESISTANCE Genes induced in DU145-RD cells (prostate cancer) with acquired resistance to docetaxel	20	-2.12	0.006
4	MELLMAN_TUT1_TARGETS_UP Genes induced in HEK293 cells (embryo kidney) after knockdown of TUT1	17	-2.04	0.019
5	**PEART_HDAC_PROLIFERATION_CLUSTER_UP** Cell proliferation genes induced by HDAC inhibitors SAHA and depsipeptide	49	-1.98	0.036
6	REACTOME_RIP_MEDIATED_NFKB_ACTIVATION_VIA_DAI Genes involved in RIP-mediated NF-kB actibation via DAI	15	-1.92	0.066
7	KEGG_GLYCOSAMINOGLYCAN_DEGRADATION Canonical pathway for degradation of glycosaminoglycans	16	-1.91	0.068
8	SABATES_COLORECTAL_ADENOMA_SIZE_UP Genes that are positively correlated with the size of colorectal adenoma	15	-1.90	0.063
9	ONDER_CDH1_SIGNALING_VIA_CTNNB1 Differentially expressed genes in HMLE cells (mammary epithelium) after RNAi of CDH1 and CTNNB1	49	-1.90	0.062
10	RIZ_ERYTHROID_DIFFERENTIATION_HEMGN Genes induced by TLX1 in iEBHX15-4 cells (pro-erythroblasts) whose expression follows that of HEMGN	15	-1.87	0.073
11	KEGG_LYSOSOME Canonical pathway for lysosome function	111	-1.84	0.109
12	WILSON_PROTEASES_AT_TUMOR_BONE_INTERFACE_UP Protease genes induced at the tumor-bone interface compared to tumor alone	15	-1.81	0.131
13	LEE_LIVER_CANCER_MYC_DN Genes suppressed in hepatocellular carcinoma by overexpression of MYC	29	-1.81	0.126
14	PID_HES_HEYPATHWAY Canonical Notch-mediated HES/HEY network	34	-1.81	0.119
15	BIOCARTA_TNFR1_PATHWAY Canonical TNFR1 signalling pathway	29	-1.81	0.113
16	SHI_SPARC_TARGETS_UP Genes induced in glioma cell lines after knockdown of SPARC by RNAi	17	-1.80	0.113
17	MIKKELSEN_IPS_HCP_WITH_H3_UNMETHYLATED Genes with high-CpG-density promoters without H3 methylation at H3K4 or H3K27 in MCV8.1 cells	21	-1.79	0.132
18	DORSEY_GAB2_TARGETS Genes induced by GAB2 in K562 cells (chronic myeloid leukemia) with p210 BCR-ABL	19	-1.78	0.120
19	WENG_POR_TARGETS_GLOBAL_DN Genes suppressed in liver from transgenic mice with reduced expression of POR in all tissues	18	-1.78	0.121
20	BUCKANOVICH_T_LYMPHOCYTE_HOMING_ON_TUMOR_DN Genes suppressed in microdissected ovarian tumors with tumor-infiltrating lymphocytes	15	-1.78	0.118
21	MIKKELSEN_MCV6_ICP_WITH_H3K27ME3 Genes with intermediate-CpG-density promoters bearing tri-methylation at H3K27 in MCV6 cells (embryonic fibroblasts)	17	-1.77	0.117
22	REACTOME_GLYCOSPHINGOLIPID_METABOLISM Genes involved in glycosphingolipid metabolism	25	-1.77	0.117
23	REACTOME_TRAF6_MEDIATED_NFKB_ACTIVATION Genes involved in TRAF6-mediated activation of NF-kB	18	-1.74	0.157
24	REACTOME_TAK1_ACTIVATES_NFKB_BY_PHOSPHORYLATION_AND_ACTIVATION_OF_IKKS_COMPLEX Genes involved in TAK1 activate NF-kB by phosphorylation and activation of IKKs complex	18	-1.73	0.159
25	CHANDRAN_METASTASIS_TOP50_UP Top 50 genes induced in metastatic vs. primary prostate cancer tumors	31	-1.73	0.163
26	REACTOME_SMOOTH_MUSCLE_CONTRACTION Genes involved in smooth muscle contraction	16	-1.72	0.172
27	EBAUER_TARGETS_OF_PAX3_FOXO1_FUSION_DN Genes suppressed in Rh4 cells (alveolar rhabdomyosarcoma) after knockdown by PAX3-FOXO1 by RNAi	26	-1.72	0.174

Gene sets (Name), number of genes (Size), normalized enrichment score (NES), and false-discovery correction (FDR) are shown for associated gene sets with FDR less than 20% that were derived from GSEA using only curated gene sets (c2). Gene sets involving histone deacetylase function are highlighted in bold.

### Histone deacetylase inhibitors regulate the pro-inflammatory response of human monocytes to in-vitro M.tb infection

**Fig 3:**
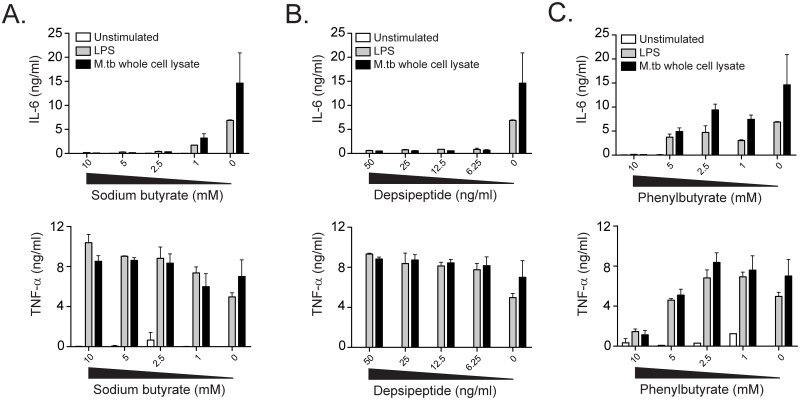
Histone deacetylase inhibitors modulate the innate immune response to M.tb. U937 cells were stimulated with media alone, LPS at a final concentration of 100 ng/ml, or M.tb whole cell lysate at a final concentration of 25 μg/ml after one hour pre-treatment with titrating doses of histone-deacetylase inhibitors. IL-6 and TNF-α were quantified in supernatants harvested after 18 hours for (A) sodium butyrate, (B), depsipeptide, and (C) phenylbutyrate. These data are representative of at least three independent experiments.

**Fig 4:**
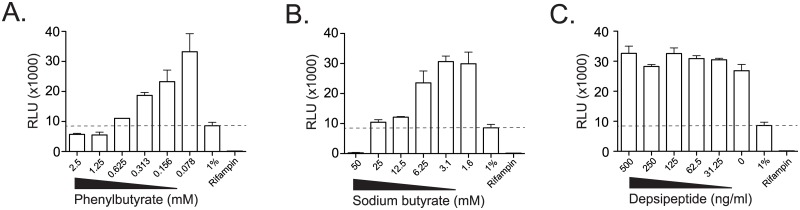
The effect of histone deacetylase inhibitors on M.tb growth. The effect of histone deacetylase inhibitors (A) depsipeptide, (B) sodium butyrate, and (C) phenylbutyrate on mycobacterial growth in broth culture was measured using an autoluminescent strain of M.tb that has been stably transfected with the bacterial Lux operon. Controls included rifampin (RIF) at a final concentration of 1 microgram/ml and 1% of the inoculum (1%) to assess the minimum inhibitory concentration 99% (MIC99—dashed line). The asterix indicates the first dose at which luminescence is not significantly different from or less than the 1% inoculum. M.tb was grown in media supplemented with titrating doses of HDACi for seven days prior to measuring relative light units (RLU). Results are representative of at least three independent experiments.

The results from GSEA include gene sets that were induced and suppressed after treatment of cells with HDACi, so the predicted direction of effect in the setting of mycobacterial infection is not clear. We hypothesized that histone deacetylase function might be important for generating an inflammatory response to *in-vitro* mycobacterial infection. Because the availability of primary cells from cases and controls were limited, we first tested this hypothesis using human cell lines. IL-6 production from U937 cells in response to stimulation with LPS or M.tb lysate was inhibited by sodium butyrate in a dose-dependent fashion (Fig 3a). Notably, TNF-α production was not inhibited, ruling out a global toxic effect of inhibiting histone deacetylase function. These results are consistent with the known anti-inflammatory effects of HDACi and inhibitory effects on IL-6 production[31,32]. To generalize these findings, we tested two other HDACi that are FDA-approved for clinical use. Depsipeptide also showed dose-dependent inhibition of IL-6 production but not TNF-α (Fig 3b). Inhibition of both IL-6 and TNF-α was seen with phenylbutyrate but only at the highest dose tested, suggesting a toxic effect of this compound (Fig 3c). To address whether HDACi have direct effects on mycobacterial growth, we performed experiments using a strain of M.tb that has been engineered to constitutively express bacterial luciferase. We found dose-dependent inhibition of M.tb growth using phenylbutyrate and observed a minimum inhibitory concentration (MIC) of 1.25mM (Fig 4a). These data are consistent with published data and validate our system[33]. We also observed some dose-dependent inhibition with sodium butyrate, but the calculated MIC was 25-50mM (Fig 4b). Finally, we observed no inhibition of growth with depsipeptide at doses that were sufficient for suppressing IL-6 production (Fig 4c). These data reveal that some clinically approved HDACi potently inhibit IL-6 production after contact with microbial products without a significant effect on mycobacterial growth.

**Fig 5:**
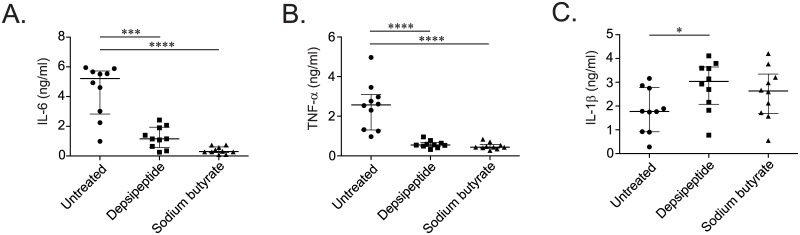
Histone-deactylase inhibitors suppress the secretion of inflammatory cytokines by primary human monocytes in response to M.tb infection. CD14+ peripheral blood monocytes from ten healthy adults were infected with M.tb strain H37Rv at a multiplicity of infection of 2.5:1 for 24 hours after one hour pre-treatment of histone deacetylase inhibitors (HDACi) depsipeptide and sodium butyrate at a final concentration of 50 ng/ml and 5mM, respectively. Cultured supernatants were tested for production of the pro-inflammatory cytokines (A) IL-6, (B) TNF-α, and (C) IL-1β. Results are representative of two replicate experiments. (*p<0.05, ***p<0.001, ****p<0.0001 by two-tailed Mann-Whitney test).

To directly investigate the link between HDACi and M.tb infection of human monocytes as suggested by our microarray data, we treated monocytes from ten healthy donors with HDACi prior to *in-vitro* infection with M.tb. Analysis of cultured supernatants after overnight incubation revealed strong inhibition of IL-6 and TNF-α production by depsipeptide and sodium butyrate (Fig 5a and 5b). Notably, IL-1β production was either not affected (sodium butyrate) or increased (depsipeptide), ruling out a toxic effect of the compounds (Fig 5c). These data support a direct role for HDACs in regulating the inflammatory response of human mononuclear phagocytes to in-vitro mycobacterial infection.

## Discussion

We compared the early transcriptional response to *in-vitro* M.tb infection in monocytes derived from M.tb-susceptible and M.tb-resistant household contacts in Uganda. We found a number of sub-cellular processes that are associated with resistance to M.tb using complementary systems biology approaches. Specifically, we show that inhibition of histone deactylase function is important for the early innate immune response to in-vitro M.tb infection. Our results demonstrate a feasible approach to understanding innate immune mechanisms underlying the phenomenon of apparent resistance to clinical M.tb infection in humans.

We identified histone deacetylase function as important for the innate immune response to *in-vitro* M.tb infection. A recent study revealed that phenylbutyrate was bacteriostatic against M.tb in culture and restricted intracellular growth in macrophages[33]. Knockdown of HDAC1 in a human macrophage cell line has been shown to reduce survival of intracellular M.tb[34]. In addition, adjunctive treatment with phenylbutyrate and Vitamin D3 were associated with higher rates of sputum culture conversion in a randomized controlled clinical trial of pulmonary tuberculosis patients[35]. Our results confirm the growth inhibitory potential of phenylbutyrate and reveal the therapeutic potential of other HDACi. In contrast to phenylbutyrate, sodium butyrate and depsipeptide potently inhibited the monocyte immune response to *in-vitro* M.tb infection without affecting growth in broth culture. Depsipeptide is structurally distinct from the butyrate compounds and was recently FDA approved for the treatment of cutaneous T-cell lymphoma[36]. As a class, butyrate compounds exhibit low-affinity interactions with HDAC proteins and have largely been surpassed by pharmacologically optimized drugs for the treatment of cancer. Our results suggest that HDACi other than phenylbutyrate may also have potential as host-directed therapies for tuberculosis. However, several aspects of our results warrant caution when considering off-label use of HDACi as host-directed therapy for clinical M.tb infection. First, we found a mostly anti-inflammatory effect in-vitro that may not predict effects in-vivo. A recent study of HDACi showed a similar discordance that did not predict increased survival in a sepsis model[37]. Second, we also found HDAC-related gene sets associated with the control subjects (gene sets 17 and 19 in S2 Table). These results highlight that the balance between infection and inflammation modulated by histone deacetylation is likely a critical determinant in clinical outcomes after clinical M.tb infection.

One limitation of our study design is that the cases we selected were younger than the control subjects, potentially confounding the association between transcriptional networks and resistance to M.tb (Table 1). However, none of the subjects in this study were younger than ten years old, and we could not discern any differences in transcriptional profiles when stratified by age (S3 Fig). It is well known that TST has decreased sensitivity in children under the age of two, but there is no evidence that this is true for older children[38]. Further, innate immune responses to inflammatory stimuli are blunted in infants and neonates but not children over five with nearly mature immune systems[39]. We have also previously shown that innate immune responses are similar between cases and controls in our cohort regardless of age[40]. Thus, it is less likely that our results are substantially confounded by age though larger studies in which samples are matched by age are required. Another potential limitation is that we cannot formally quantify the exposure of individuals who failed to convert their TST. However, we previously demonstrated no difference in a validated epidemiologic risk score between controls and cases subjects in our cohort[21]. The specific subjects studied here all showed comparable levels of high exposure to M.tb (Table 1). Our study involved hundreds of household contacts of tuberculosis cases that were followed longitudinally for two years, but the TST is at best an imperfect assessment of the presence of viable mycobacteria in the host. These results suggest that future studies should include an additional measure of clinical infection, such as IGRA, and more than two years of follow-up would make our clinical definition even more rigorous. Finally, we were unable to use GSEA to validate a hypothesis about the role of HDACi, and a small sample size precluded the use of other pathway topology or machine learning approaches might have provided complementary results. Future experiments with larger sample sizes are required to confirm and extend the statistical associations reported here.

Studying the cellular mechanisms underlying apparent clinical resistance to M.tb infection has been a major advance for the field. By combining rigorous case definitions with longitudinal follow-up, hypothesis-generating systems biology approaches, and hypothesis-testing laboratory experiments, we were able to identify the central role of HDAC function in the innate immune response to M.tb. Our data suggest that a balanced inflammatory response to initial infection with M.tb may influence long-term clinical outcomes. We expect that further laboratory investigation of differentially expressed gene sets will uncover additional mechanisms that might translate into therapies for tuberculosis as well as identifying biomarkers of heightened resistance to M.tb infection.

## Supporting information

S1 Fig PCA reveals batch effect. (PDF)

S2 Fig Results of Signaling Pathway Interaction Analysis (SPIA). (PDF)

S3 Fig Principal components plot stratified by age and treatment. (PDF)

S1 Table GSEA results including samples that were previously discarded due to batch effect. (DOCX)

S2 Table Gene sets preferentially associated with susceptible controls who develop latent tuberculosis infection. (DOCX)

S1 Methods Supplementary methods. (DOC)
