# Supplementary material for: Transcriptional networks are associated with resistance to Mycobacterium tuberculosis infection
Source: PLoS One. 2017 Apr 17;12(4):e0175844. doi: 10.1371/journal.pone.0175844 (PMC5393882; doi:10.1371/journal.pone.0175844)
Supplement: S1 Fig — (PDF) [file pone.0175844.s001.pdf]

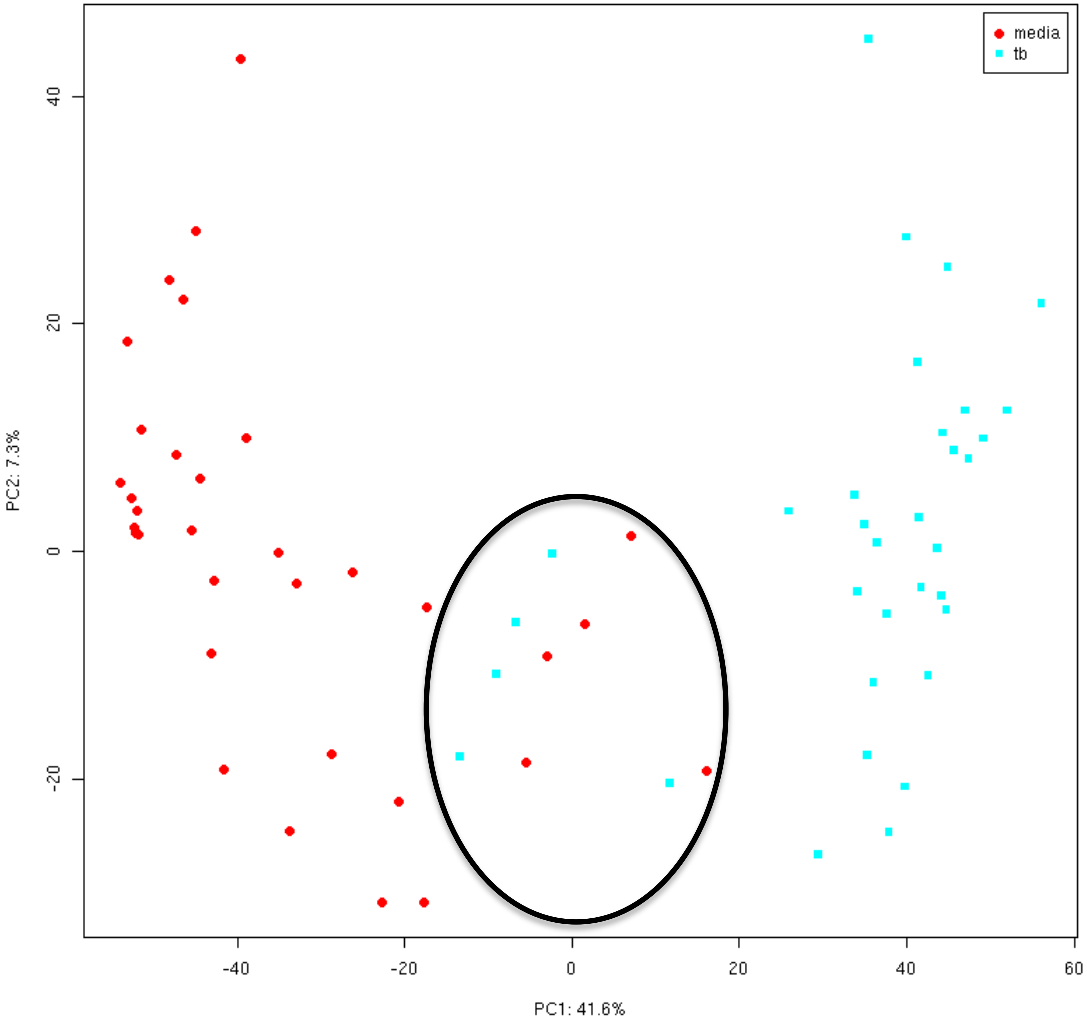


**S1 Figure. PCA Reveals Batch Effect.** Principal components analysis of the full dataset of 13 TSTNEG and 20 TSTPOS subjects revealed ten outlier samples that are poorly resolved by PC1 (black circle). These ten samples are pairs (media or M.tb treated) from five individuals (3 TSTNEG and 2 TSTPOS) that were processed on the same day. Because of the obvious batch effect, these samples were removed and not included in the final dataset.
