# Supplementary material for: Transcriptional networks are associated with resistance to Mycobacterium tuberculosis infection
Source: PLoS One. 2017 Apr 17;12(4):e0175844. doi: 10.1371/journal.pone.0175844 (PMC5393882; doi:10.1371/journal.pone.0175844)
Supplement: S2 Fig — (PDF) [file pone.0175844.s002.pdf]

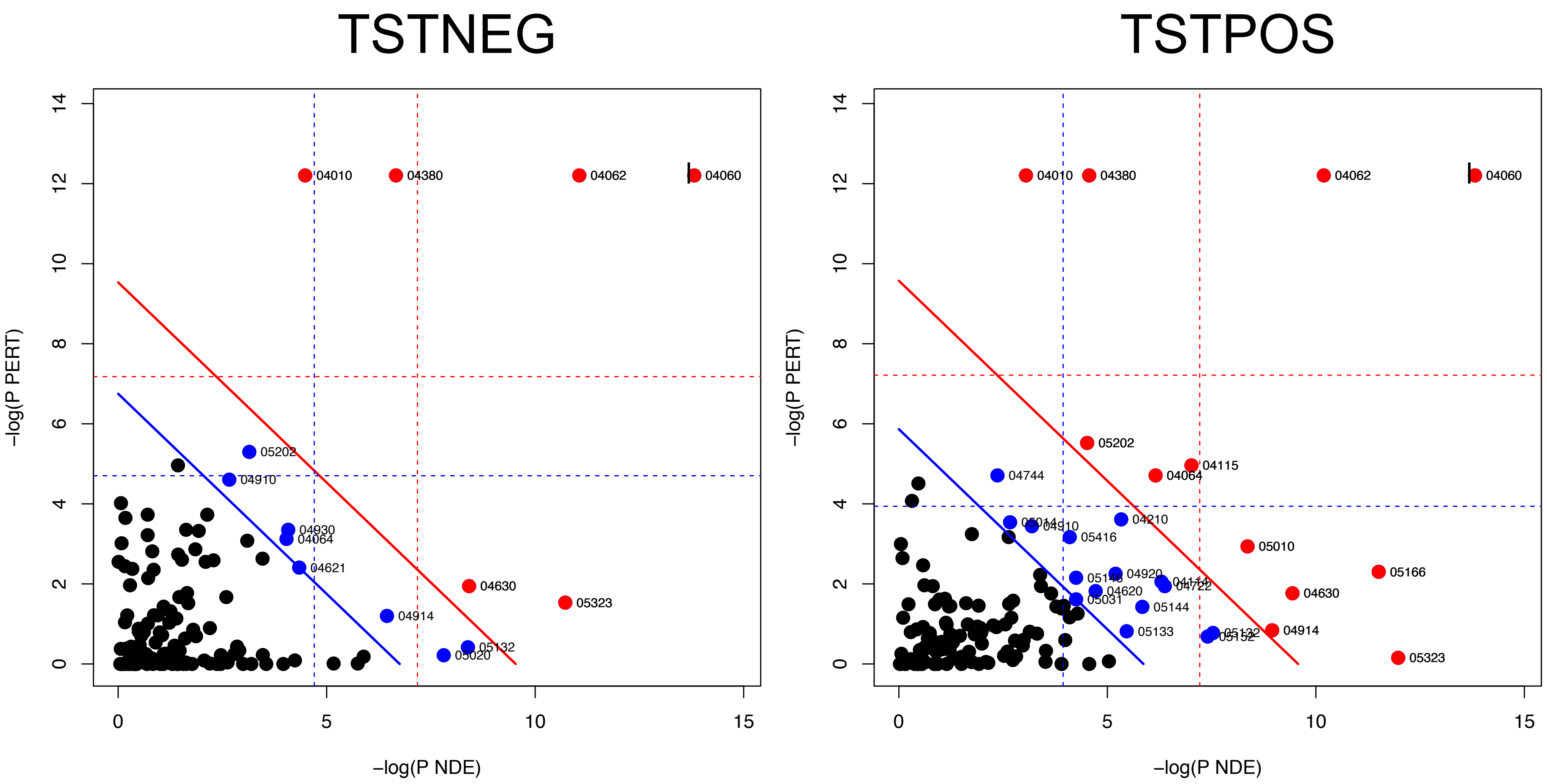

S2 Figure. Results of Signaling Pathway Interaction Analysis (SPIA). TSTNEG and TSTPOS samples were analyzed independently by SPIA. Within the TSTPOS data there are twelve KEGG pathways that meet pre-defined criteria for significant perturbation. Six of these pathways are also significant in TSTNEG data. KEGG pathways are indicated by their numbers Instead of their names. P NDE reflects the probability that the number of differentially expressed genes is over-represented in a given pathway. P PERT reflects the amount of perturbation that is measured in each pathway and accounts for topology or hierarchy of biological networks.
