# Supplementary material for: Transcriptional networks are associated with resistance to Mycobacterium tuberculosis infection
Source: PLoS One. 2017 Apr 17;12(4):e0175844. doi: 10.1371/journal.pone.0175844 (PMC5393882; doi:10.1371/journal.pone.0175844)
Supplement: S3 Fig — (PDF) [file pone.0175844.s003.pdf]

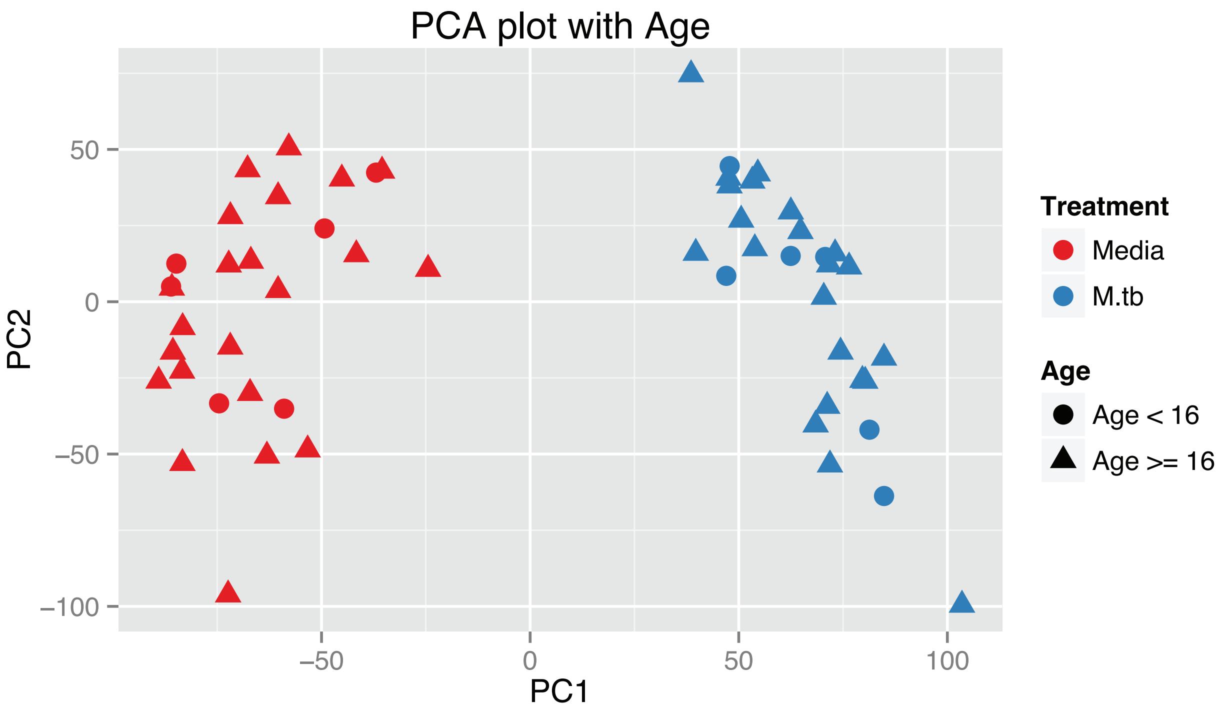

Supplementary Figure 3. Principal Components Plot stratified by Age and Treatment. The data shown in Figure 1B of the main text was re-labelled using age instead of TST status. Samples arising from 6 study subjects under the age of 16 are evenly distributed among those greater than 16 years of age
