# Supplementary material for: Transcriptional networks are associated with resistance to Mycobacterium tuberculosis infection
Source: PLoS One. 2017 Apr 17;12(4):e0175844. doi: 10.1371/journal.pone.0175844 (PMC5393882; doi:10.1371/journal.pone.0175844)
Supplement: S1 Table — (DOCX) [file pone.0175844.s004.docx]

| **Rank** | **NAME** | **FDR** | **Previous Rank** |
| --- | --- | --- | --- |
| 1 | MOOTHA_TCA | 0.052 |  |
| 2 | EBAUER_TARGETS_OF_PAX3_FOXO1_FUSION_DN | 0.042 | 27 |
| 3 | JOSEPH_RESPONSE_TO_SODIUM_BUTYRATE_DN | 0.063 | 1 |
| 4 | REACTOME_TRAF6_MEDIATED_NFKB_ACTIVATION | 0.070 | 23 |
| 5 | PATTERSON_DOCETAXEL_RESISTANCE | 0.056 | 3 |
| 6 | PID_ARF6_PATHWAY | 0.056 |  |
| 7 | LEE_LIVER_CANCER | 0.142 | 13 |
| 8 | KYNG_RESPONSE_TO_H2O2_VIA_ERCC6_UP | 0.158 |  |
| 9 | REACTOME_SMOOTH_MUSCLE_CONTRACTION | 0.144 | 26 |
| 10 | YAO_TEMPORAL_RESPONSE_TO_PROGESTERONE_CLUSTER_6 | 0.204 |  |
| 11 | KAMMINGA_SENESCENCE | 0.192 |  |
| 12 | MEISSNER_NPC_HCP_WITH_H3K4ME3_AND_H3K27ME3 | 0.185 |  |
| 13 | ZHAN_MULTIPLE_MYELOMA_CD1_AND_CD2_DN | 0.172 |  |
| 14 | PID_S1P_S1P1_PATHWAY | 0.228 |  |
| 15 | BIOCARTA_VEGF_PATHWAY | 0.229 |  |
| 16 | CHIANG_LIVER_CANCER_SUBCLASS_POLYSOMY7_UP | 0.228 |  |
| 17 | LIU_LIVER_CANCER | 0.215 |  |
| 18 | CAMPS_COLON_CANCER_COPY_NUMBER_DN | 0.208 |  |
| 19 | MASSARWEH_RESPONSE_TO_ESTRADIOL | 0.210 |  |
| 20 | LANDIS_ERBB2_BREAST_TUMORS_65_DN | 0.205 |  |
| 21 | REACTOME_CIRCADIAN_REPRESSION_OF_EXPRESSION_BY_REV_ERBA | 0.211 |  |
| 22 | LEE_METASTASIS_AND_ALTERNATIVE_SPLICING_UP | 0.205 |  |
| 23 | WANG_BARRETTS_ESOPHAGUS_UP | 0.201 |  |
| 24 | MOOTHA_GLUCONEOGENESIS | 0.203 |  |
| 25 | REACTOME_GLYCOSPHINGOLIPID_METABOLISM | 0.227 | 22 |
| 26 | TONG_INTERACT_WITH_PTTG1 | 0.248 |  |

**S1 Table. GSEA results including samples that were previously discarded due to batch effect**. Paired (M.tb-treated or media treated) microarrays from 20 TSTPOS and 12 TSTNEG subjects were pre-processed and analyzed in GSEA as described in the Methods. Shown here are gene sets that were enriched among TSTNEG with FDR < 25%. Also noted are gene sets that appear in Table 2 of the original manuscript and previous rank in that list.
