# Supplementary material for: Transcriptional networks are associated with resistance to Mycobacterium tuberculosis infection
Source: PLoS One. 2017 Apr 17;12(4):e0175844. doi: 10.1371/journal.pone.0175844 (PMC5393882; doi:10.1371/journal.pone.0175844)
Supplement: S2 Table — (DOCX) [file pone.0175844.s005.docx]

**S2** **Table. Gene sets preferentially associated with susceptible controls who develop latent tuberculosis infection**. Gene sets (Name), number of genes (Size), normalized enrichment score (NES), and false-discovery correction (FDR) are shown for associated gene sets with FDR less than 20% that were derived from GSEA using only curated gene sets (c2). Gene sets involving histone deacetylase function are highlighted in bold.

| **RANK** | **NAME** | **SIZE** | **NES** | **FDR** |
| --- | --- | --- | --- | --- |
| 1 | GEORGANTAS_HSC_MARKERS | 46 | 2.27 | 0.005 |
| 2 | YANG_BREAST_CANCER_ESR1_LASER_UP | 19 | 2.21 | 0.004 |
| 3 | CHESLER_BRAIN_HIGHEST_GENETIC_VARIANCE | 26 | 2.10 | 0.015 |
| 4 | PID_TAP63PATHWAY | 38 | 2.05 | 0.030 |
| 5 | ST_PHOSPHOINOSITIDE_3_KINASE_PATHWAY | 35 | 2.03 | 0.030 |
| 6 | LI_LUNG_CANCER | 31 | 1.99 | 0.041 |
| 7 | REACTOME_RNA_POL_I_PROMOTER_OPENING | 30 | 1.98 | 0.046 |
| 8 | BIOCARTA_IL2_PATHWAY | 20 | 1.97 | 0.045 |
| 9 | KAPOSI_LIVER_CANCER_MET_UP | 15 | 1.96 | 0.046 |
| 10 | BIOCARTA_EPO_PATHWAY | 18 | 1.92 | 0.063 |
| 11 | BIOCARTA_P53HYPOXIA_PATHWAY | 20 | 1.87 | 0.105 |
| 12 | GENTLES_LEUKEMIC_STEM_CELL_UP | 18 | 1.86 | 0.106 |
| 13 | REACTOME_RESPIRATORY_ELECTRON_TRANSPORT_ATP_SYNTHESIS_BY_CHEMIOSMOTIC_COUPLING_AND_HEAT_PRODUCTION_BY_UNCOUPLING_PROTEINS_ | 69 | 1.84 | 0.130 |
| 14 | REACTOME_RESPIRATORY_ELECTRON_TRANSPORT | 56 | 1.84 | 0.121 |
| 15 | TIMOFEEVA_GROWTH_STRESS_VIA_STAT1_DN | 15 | 1.83 | 0.124 |
| 16 | **DELLA_RESPONSE_TO_TSA_AND_BUTYRATE** | 20 | 1.81 | 0.148 |
| 17 | REACTOME_RNA_POL_I_TRANSCRIPTION | 52 | 1.81 | 0.142 |
| 18 | **PID_HDAC_CLASSII_PATHWAY** | 28 | 1.80 | 0.145 |
| 19 | SPIELMAN_LYMPHOBLAST_EUROPEAN_VS_ASIAN_2FC_DN | 16 | 1.80 | 0.139 |
| 20 | SCHLOSSER_SERUM_RESPONSE_AUGMENTED_BY_MYC | 84 | 1.79 | 0.149 |
| 21 | WAKABAYASHI_ADIPOGENESIS_PPARG_BOUND_36HR | 25 | 1.78 | 0.149 |
| 22 | REACTOME_AMYLOIDS | 40 | 1.78 | 0.150 |
| 23 | LIM_MAMMARY_LUMINAL_MATURE_DN | 57 | 1.76 | 0.186 |
| 24 | NOJIMA_SFRP2_TARGETS_DN | 20 | 1.75 | 0.187 |
