# Supplementary material for: Transcriptional networks are associated with resistance to Mycobacterium tuberculosis infection
Source: PLoS One. 2017 Apr 17;12(4):e0175844. doi: 10.1371/journal.pone.0175844 (PMC5393882; doi:10.1371/journal.pone.0175844)
Supplement: S1 Methods — (DOC) [file pone.0175844.s006.doc]

**Supplementary Methods**

*Microarray Data Processing*

Probe intensities were quantified using Illumina iScan platform and processed using BeadArray without performing normalization or background correction. Subsequent data processing was performed in the R Statistical Environment using the ‘lumi’ package (17). Data were quantile normalized, log2 transformed, and divided into four subsets: TSTPOS-Media, TSTPOS-M.tb, TSTNEG-Media, and TSTNEG-M.tb. These four data sets were used to perform SPIA. A signal intensity threshold was defined as the 75% percentile of the fluorescence intensity of negative control probes. The signal intensity filter was applied such that for each probe at least 75% of the samples in a subset must have expression above the threshold to pass the filter. After signal intensity filtering, probes were collapsed to genes using the absolute value of the maximum expression among multiple probes for a given gene. Finally, log transformed media control values were subtracted from M.tb values yielding fold-changes for each gene. In the discovery analysis, 48,804 probes were filtered and collapsed to 13,970 genes for analysis with Limma and GSEA. Detailed R code is available at <https://github.com/seshadrilab>.

*Microarray Data Analysis*

Single gene comparisons between the two groups were performed with Limma and adjusted for multiple-hypothesis testing using the Benjamini-Hochberg method. Signaling pathway interaction analysis was performed with the SPIA package in R with the following defaults: 2000 bootstrap iterations, Fisher’s method to combine p-values, and NULL value for beta SPIA was run separately for cases and controls examining genes that were differentially expressed between M.tb and Media with a false discovery value of 10%. Gene Set Enrichment Analysis (GSEA) was performed with the collapsed dataset described above upon calling the Java applet from the Broad Institute server on August 6, 2014. The curated gene set ‘c2.all.v4.0.symbols.gmt’ and immunological signatures set ‘c7.all.v4.0.symbols.gmt’ from The Molecular Signatures Database (MSigDB) were queried for associations. Default GSEA parameters were used except for the following: metric (Signal2Noise), set_min (15), and nperm (1000) and randomization (equalize and balance). We performed a sensitivity analysis of the GSEA algorithm by varying these parameters. We found that the rank order of results shown in Table 2 was minimally altered. Importantly, both ‘JOSEPH’ and ‘PEART_HDAC’ gene sets were still top hits.
